# EHMTI-0118. Risk factors of migraine - a co-twin control study

**DOI:** 10.1186/1129-2377-15-S1-G14

**Published:** 2014-09-18

**Authors:** HD Hambardzumyan, HM Manvelyan

**Affiliations:** 1 Neurology, Yerevan State Medical University, Yerevan, Armenia

## Introduction

Migraine is a very frequent disease in Armenia.

## Aims

To evaluate the effect of a variety of diseases and environmental risk factors on the risk of migraine.

## Methods

In 2012, more than 10.000 twin individuals participated in a questionnaire survey. Only complete same-sexed twin pairs discordant for migraine were included in this co-twin control study, which comprised 554 female and 325 male pairs. This design allowed us to control for most shared genetic and environmental predisposition.

## Results

Low back pain, neck pain and whiplash were significantly associated with migraine with an increased risk of 50-110%. Arterial hypertension, kidney stone, osteoarthritis and tinnitus were also significantly associated with migraine. Coronary thrombosis and other thrombosis as separate entities were not associated with migraine, however, when pooled together there was significantly increased risk(OR=1.88[1.14-3.08], p=0.01). Weekly alcohol consumption was the only environmental factor significantly associated with migraine. The risk was decreased with almost 20%.The effect of obesity on the risk of migraine was 1.5, however, this was not statistically significant(p=0.07).

## Conclusions

This is the first large co-twin study of co-morbidities and environmental risk factors in migraine. A number of co-morbidities were confirmed but no environmental risk factors besides alcohol consumption were significant. Alcohol is a potent inducer of migraine attack therefore the association between alcohol and migraine is likely due to an avoidance reaction towards alcohol. Conventional socio-economic and lifestyle factors therefore do not seem to be important. Further studies should focus on other factors, e.g. emotional factors such as stressful events.

No conflict of interest.

